# Relationship of Serum Vitamin D Concentrations and Allostatic Load as a Measure of Cumulative Biological Risk among the US Population: A Cross-Sectional Study

**DOI:** 10.1371/journal.pone.0139217

**Published:** 2015-10-09

**Authors:** Regina Frei, Sarah R. Haile, Margot Mutsch, Sabine Rohrmann

**Affiliations:** Division of Chronic Disease Epidemiology, Epidemiology, Biostatistics and Prevention Institute (EBPI), University of Zurich, Zurich, Switzerland; Indiana University, UNITED STATES

## Abstract

**Introduction:**

The allostatic load (AL) index is a multi-systemic measure of physiologic dysregulation known to be associated with chronic exposure to stress and adverse health outcomes. We examined the relationship between AL and serum 25-hydroxyvitamin D (25(OH)D) concentration in non-institutionalized US adults.

**Methods:**

Data from the Third National Health and Nutrition Examination Survey (NHANES III, 1988–94) were used to calculate two versions of AL including 9 biomarkers and another two with 14 biomarkers (systolic and diastolic blood pressure, pulse rate, serum cholesterol, serum HDL-cholesterol, glycated hemoglobin, sex-specific waist-to-hip ratio, serum albumin, and serum C-reactive protein for AL1, and, additionally body mass index, serum triglyceride, serum creatinine, and serum herpes I & II antibodies for AL2), each set defined by predefined cut-offs or by quartiles. Serum vitamin D concentration was ranked into quartiles. Logistic regression, Poisson regression and linear regression were used to examine the association of serum 25(OH)D concentrations on AL, after adjusting for biological, physiological, socioeconomic, lifestyle, and health variables.

**Results:**

Odds Ratios (OR) for high AL of the lowest 25(OH)D serum quartile were between 1.45 (95% CI: 1.28, 1.67) and 1.79 (95% CI: 1.39, 2.32) for the fully adjusted model, depending on AL version. Inverse relationships between vitamin D serum concentrations were observed for all AL versions and every adjustment. This relationship was consistent after stratification by sex, age or ethnic background. Sensitivity to low 25(OH)D concentrations was highest among the youngest group (20–39 years) with an OR of 2.11 (95% CI: 1.63, 2.73) for the lowest vitamin D quartile Q1.

**Conclusions:**

Vitamin D had a consistent and statistically significant inverse association with all tested models of high AL, which remained consistent after adjusting for biological, socioeconomic, lifestyle and health variables. Our study adds evidence linking low 25(OH)D concentrations with poorer health, further-reaching than bone health.

## Introduction

Chronic stress, such as repeated exposure to stressors–or exposure over an extended time period–is known to be associated with negative effects on human health [1]. Thereby, chronic stress has been linked with health conditions such as cardiovascular disease (CVD), metabolic, immunologic, psychological and cognitive disorders [2–5]. The universal use of the term “stress” in popular language makes it an imprecise word to describe how the body copes with changing environmental, psychosocial and physical circumstances. In order to find a more comprehensive and process-oriented term, “allostasis” was established, meaning literally “maintaining stability through change”. The allostasis model suggests that the goal of regulation is resilience to adapt to environmental demands [6]. To match physiological parameters of chronic stress with allostasis, the allostatic load (AL) index was introduced by McEwen and Stellar [1]. AL is a cumulative measure of biomarkers representing physiological stress in the organism, such as neuroendocrine, cardiovascular, immune and metabolic biomarkers [7]. High AL has been linked with several adverse health outcomes including all-cause mortality [7–9], cardiovascular diseases [7, 10], and diminished mental health [11].

The AL index was originally measured using 10 biomarkers: systolic and diastolic blood pressure, total cholesterol (TC), high-density-lipoprotein (HDL), glycated hemoglobin (HbA1c), waist-to-hip ratio (WHR), dehydroepiandrosterone sulfate (DHEA-S), urinary epinephrine, norepinephrine, and cortisol [7]. The biomarkers were dichotomized with the highest quartile coding for high risk (lowest quartile for DHEA-S and HDL) and summed for the AL index. More recent studies used variable numbers of biomarkers and different approaches to dichotomization [12, 13]. Currently, there is no consensus on either the choice of or method for dichotomizing the biomarkers which comprise the AL index.

Various studies have linked AL with social contexts, including socioeconomic status [14, 15], ethnicity [8, 16, 17], neighborhood [17, 18], gender [19], and social relationships [20]. Rosenberg et al. found an association between AL and serum carotenoid concentration among middle-aged US adults [21]. However, there is limited knowledge on the impact of lifestyle factors, especially nutrition, on AL.

Discussion of vitamin D has expanded considerably since studies have found its role far beyond bone health. Recent studies have focused on the impact of 25(OH)D on the immune response, respiratory health, diabetes risk, cancer, CVDs or obesity, as presented in several systematic reviews and meta-analyses [22–24].

According to Troesch et al., who analyzed the vitamin intake in several industrialized countries (including the US, Germany, UK, and the Netherlands), vitamin D is one of the critical vitamins for which intake is often below the recommended level [25]. Globally, 6.7% of the overall population is reported to be vitamin D deficient (concentration below 25 nmol/L), 37% have estimates below 50 nmol/L [26].

After vitamin D is photosynthesized in the skin or ingested via food, it is hydroxylated in the liver to 25(OH)D (calcifediol) and then transported to the kidneys, where it is hydroxylated to 1,25(OH)2D (calcitriol), the active metabolite [27]. 1,25(OH)2D is a potent steroid hormone that regulates the immune system via the vitamin D receptor (VDR) which is present in many immune cell types such as monocytes, macrophages and dendritic cells [28]. In general, 1,25(OH)2D activates the innate immune system and inhibits adaptive immunity, and as a result it may serve a critical role in coordinating inflammatory and anti-inflammatory processes [29, 30]. Due to the interrelation of chronic stress, immune response, and inflammation, it has been suggested that sufficient concentrations of vitamin D may protect against chronic stress, assessed with a high AL index as a marker. In this cross-sectional study, we therefore aimed to investigate the relationship between AL and serum 25(OH)D concentrations. Based on our findings, we discuss the implications for the reduction of stress-related adverse health outcomes.

## Methods

### Allostatic Load

Allostatic load was calculated as a summary measure of multisystem dysregulation including cardiovascular, metabolic and inflammation-related biomarkers. We defined two AL scores: allostatic load 1 (AL1) calculated from 9 biomarkers and allostatic load 2 (AL2) calculated from 14 biomarkers. Because different methods for low/high risk calculation of biomarkers were used in previous studies, we assessed our biomarkers in two ways, once using predefined cut points and once analyzing the AL counts in quartiles (Table 1). This leads to four AL versions for this study: AL1 and AL2 calculated with the cut point method and AL1 and AL2 calculated with the quartile method.

**Table 1:** Cut points for biomarkers and prevalence of high-risk biomarkers with cut point and quartile method.

AL version	Biomarker	Cut point value	Prevalence of high-risk with cut point calculation [%]	Prevalence of high-risk with quartile calculation [%]	Correlation with 25(OH)D
**AL1 [8, 14, 17, 21]**	Albumin [g/L]	< 38	10.4	24.3	0.14
	C-reactive protein [mg/dl]	≥ 0.3	27.9	20.1	-0.07
	Waist-to-hip ratio	> 0.9 for men; > 0.85 for women	62.7	18.8	-0.06
	Total cholesterol [mg/dl]	≥ 240	19.1	23.5	-0.03
	HDL cholesterol [mg/dl]	< 40	21.1	26.2	0.03
	Glycated hemoglobin [%]	≥ 6.4	5.4	15.3	-0.14
	Resting heart rate [bt/min]	≥ 90	10.0	23.8	-0.08
	Systolic blood pressure [mm Hg]	≥ 140	14.8	17.7	-0.10
	Diastolic blood pressure [mm Hg]	≥ 90	6.4	24.4	-0.06
**Additional biomarkers for AL2**	Body mass index [kg/m^2^] [31]	≥ 30	20.2	20.8	-0.23
	Creatinine [mg/dl] [33]	≥ 1.4	5.5	26.1	0.01
	Triglyceride [mg/dl] [32]	≥ 150	26.4	22.3	-0.10
	Herpes I antibody	pos/neg	67.0	67.0	0.12
	Herpes II antibody	pos/neg	23.6	23.6	0.12

AL, allostatic load.

**Fig 1:**
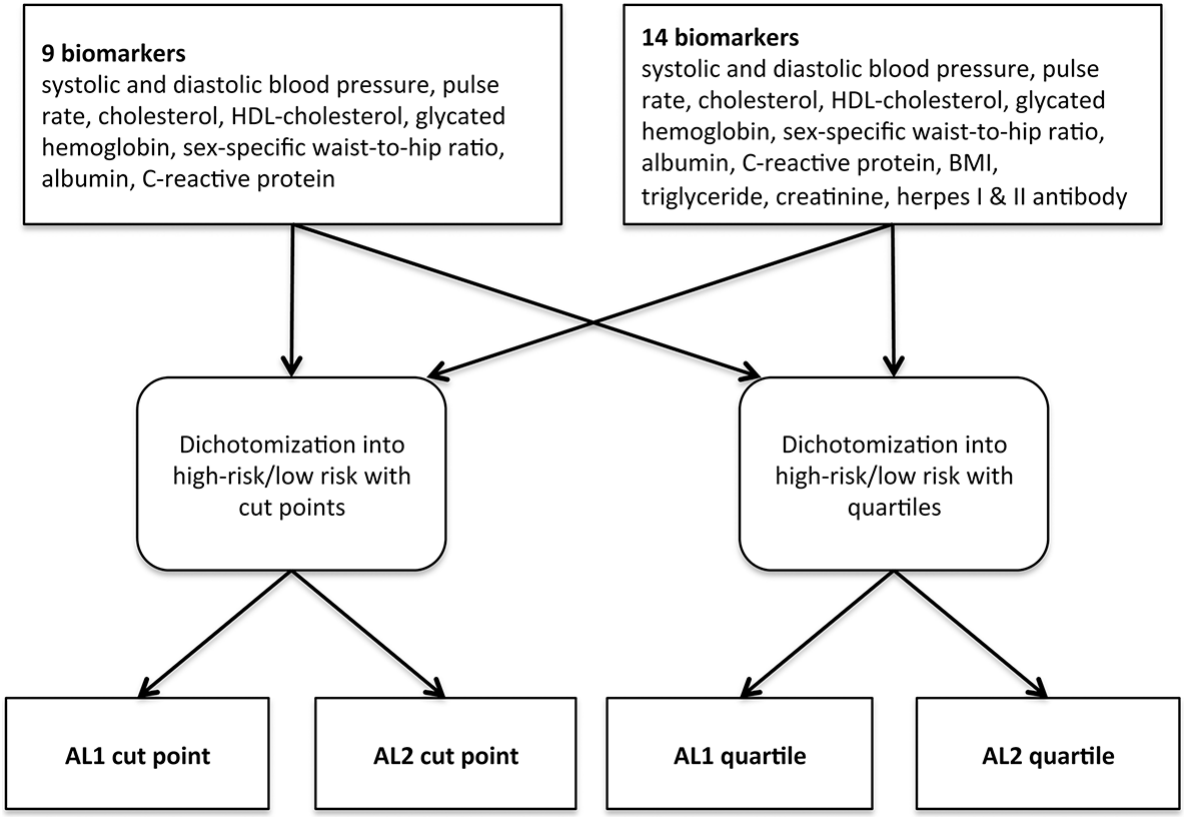
Procedure to establish the four allostatic load versions. AL, allostatic load index, BMI, body mass index, HDL-cholesterol, high-density lipoprotein-cholesterol.

In AL1, we included systolic and diastolic blood pressure, pulse rate, serum cholesterol, serum HDL, glycated hemoglobin, WHR, serum albumin, and serum C-reactive protein (CRP) as it was done previously [14, 15]. For AL2, we additionally included body mass index (BMI) [31], serum triglyceride (TG) [32], serum creatinine [33], and serum herpes simplex virus I & II antibodies (H1A & H2A). Fig 1 provides an overview. Each of the biomarkers was dichotomized into “high risk = 1” and “low risk = 0”. The dichotomization was done with cut points, as shown in Table 1, or with quartiles, respectively. For the quartiles, the highest quartile coded for high risk for all biomarkers except albumin and HDL-cholesterol, where the lowest quartile represented high risk. Serum herpes I & II antibodies were binary variables with positive indicating as “high risk”. AL score-ranges were 0–9 for AL1 and 0–14 for AL2. For all four versions of AL, summary scores of 0 to 2 were defined as “low AL” and ≥3 as “high AL”, as done in previous studies [8, 16, 20].

### Study Population

The data for the current study originates from the Third National Health and Nutrition Examination Survey (NHANES III, 1988–1994). NHANES is a cross-sectional study providing information on the distribution of various health and nutritional indicators and potential risk factors. The survey is representative of the non-institutionalized US population, with oversampling of African-Americans, Mexican-Americans and people aged 60 years and over. Additional information about NHANES III can be found elsewhere [34, 35].

The original sample size for this study was 20,050. Participants younger than 20 years (n = 1,225) or without valid serum vitamin D data (n = 3,076) were excluded. Furthermore, participants with missing data for any of the biological markers used for the calculation of the AL (n = 4,844 for AL1 and n = 15,057 for AL2) were excluded. After missing data exclusions, data from 14,213 subjects, (7,510 females) were available for calculating AL1 and 4,620 subjects (2,334 females) available for AL2. Some participants have more than one missing value(for example one is under 20 years old and has a missing value for 25(OH)D shows up in both "missing numbers"), and therefore subtraction leads to other results.

### Serum Vitamin D 25(OH)D

Concentrations of serum vitamin D 25(OH)D were measured with a radioimmunoassay kit (DiaSorin, Inc., Stillwater, MN; normal range 22.5–94 nmol/L). Blood samples were collected in northern latitudes during summer and in southern latitudes during winter.

In the absence of a generally accepted definition of 25(OH)D deficiency, most investigators assessed serum concentrations higher than 50 nmol/L as sufficient [36]. Serum concentrations under 25 nmol/L have been associated with disorders of bone metabolism [26, 36]. Therefore, we used 25 nmol/L as a cut point for severe vitamin D deficiency and 50 nmol/L for moderate vitamin D deficiency. Furthermore, vitamin D serum concentrations were categorized into quartiles based on the distribution in the cohort with quartile 1 (Q1) as the lowest vitamin D concentration. We defined vitamin D supplementation as consumption of any vitamin D containing supplement for at least one month on at least 20 days per month.

### Covariates

Covariates included biological variables (age, sex, race/ethnicity), socioeconomic variables (education, census region, urbanization, marital status, poverty-income ratio), lifestyle factors (alcohol consumption, smoking status, physical activity, diet) and health variables (self-reported general health, long-term medication). Age in years was included as a continuous factor. Race/ethnicity categories were non-Hispanic White, non-Hispanic Black, Mexican-American and other. Education was classified based on US Census criteria in “less than high-school”, “high-school graduate” and “college graduate”. Living environment was defined both geographically (Northeast, Midwest, South and West) and by type (i.e., as urban (central counties of metro areas with a population of 1 million or more) or rural). Marital status was coded as “married or living as married”, “widowed, divorced or separated” and “never married”. Poverty-income ratio is the ratio of the midpoint of the observed family income as the numerator and the poverty threshold, the age of the family reference person, and the calendar year in which the family was interviewed as the denominator. According to the NHANES III recommendation, poverty-income ratios below 1 were coded as “below poverty” and 1 and above as “at or above poverty” [35]. Alcohol consumption was based on participants’ statements about frequency of beer, wine or hard liquor intake, and was grouped into sex-adjusted categories of “non-drinker”, “moderate-drinker” or “excessive-drinker”, as described elsewhere [37]. The healthy-eating index is a measure of overall dietary quality and was treated as a continuous variable, as described elsewhere [38]. Smoking behavior was determined by questionnaire and grouped into “non-smoker”, “current-smoker” or “ex-smoker”. The frequency of moderate and vigorous physical activity was grouped into age-adjusted categories of “no activities”, “1–2 activities per week”, “3–4 activities per week” and “5+ activities per week”. Self-reported general health was determined by questionnaire and included “excellent”, “very good and good” and “fair and poor”. Long-term medications, including systemic drugs such as antihypertensives, antihyperlipidemics, blood glucose regulators, mental health medications, antineoplastics and others, were counted (“medication”) if they had been taken for more than 90 days, otherwise it was considered “no medication”.

### Statistical Analyses

The current study results were weighted for the complex multistage probability sampling design and population estimation, with weighting factors provided by NHANES III, in order to account for the complex survey design, survey non-response, and post-stratification. Logistic regression was used to calculate the relationship of AL (high versus low) with individual serum vitamin D. AL could also be considered a count of the corresponding biomarkers, which was analyzed using Poisson regression. The results from zero-inflated Poisson regression, allowing more frequent 0 counts than otherwise expected, were similar to those of the standard Poisson regression, and are not shown. A further analysis considered AL as a continuous outcome examined using linear regression (S1 Table). Vitamin D was considered both as quartiles and as a continuous predictor. A basic model without adjustments was performed; further models adjusted in a nested fashion for biological factors, socioeconomic factors, lifestyle factors, and the final model additionally adjusted for health factors. All models were assessed for AL1 and AL2, both for the cut point definition and for the quartile definition. The association of vitamin D supplementation and AL as well as serum vitamin D concentration was examined using the χ2 test. Correlations between vitamin D and the individual biomarkers were estimated using the square root of the R^2^ statistic from the corresponding linear regression models. Analyses were performed using IBM SPSS Statistics Version 22. Linear and (zero inflated) Poisson regression analyses were performed using R (version 3.2.1).

## Results

Vitamin D serum concentrations ranged from 8.7 to 243.6 nmol/L with a mean concentration of 73.7 nmol/L. The mean among male participants was 77.6 nmol/L, females had a significantly lower mean concentration of 70.2 nmol/L (p<0.001). In our sample, 2.0% of all participants had a severe deficiency in 25(OH)D concentration and 22.5% a moderate deficiency.

AL scores ranged from 0–9 for AL1 with mean values of 1.8 (standard deviation SD: 1.44) for the cut point method, 1.9 (SD: 1.68) for the quartile method. Due to the additional biomarkers, AL scores ranged from 0–14 for AL2, with mean values of 3.0 (SD: 2.06) for the AL2 cut point method and 3.3 (SD: 2.30) for the quartile method, respectively. The prevalence of a high AL was 28.6% (32.5%) for AL1 cut point (quartile) and expectedly higher at 52.8% (57.2%) for AL2 cut point (quartile).

The high-risk prevalence of individual biomarkers according to the categorization method differed for some biomarkers between the methods used (Table 1). About 25% of participants (deviations due to weighting) were in the high-risk group of glycated hemoglobin, diastolic blood pressure, and serum creatinine, but only 5.4%, 6.4%, and 5.5%, respectively, based on the cut point method. On the other hand, 62.7% were in the high-risk group for WHR when using sex-specific cut points compared with approximately 25% using the quartile method. The highest correlations between vitamin D and individual biomarkers were found for glycated hemoglobin, albumin, body mass index and antibodies against herpes simplex viruses (Table 1).

A statistically significant inverse relationship was observed between vitamin D supplementation and 25(OH)D deficiency (p<0.001, chi-square test). However, there was no statistically significant association between vitamin D supplementation and AL1 cut point (p = 0.121) or AL1 quartile (p = 0.050).

Table 2 shows the baseline characteristics by AL (AL1 cut point) and vitamin D quartiles. The distribution towards the highest quartile for both sexes was caused by the NHANES III weight, which shifted more than 25% of participants into the highest vitamin D quartile. Ethnic background was strongly associated with vitamin D quartiles, as only 9.3% non-Hispanic White participants were in the lowest quartile, but 49.4% of non-Hispanic Blacks and 22.3% of Mexican-Americans. Higher education, urban living environment and better economic status were associated with lower AL and higher serum 25(OH)D concentrations. Non-smokers, moderate-drinkers and physically active participants had the lowest percentage of high AL, whereas ex-smokers, moderate drinkers and physically active participants had the highest percentage in the highest vitamin D quartile. Participants considering themselves having excellent general health and those who took no longterm medication had lower AL and higher 25(OH)D serum concentrations.

**Table 2:** Baseline characteristics by AL1 cut point and 25(OH)D concentration quartiles.

		Categorical						Continuous	
		allostatic load		Vitamin D				allostatic load	Vitamin D
		Low [%]	High [%]	Q1 [%]	Q2 [%]	Q3 [%]	Q4 [%]	mean AL	mean 25(OH)D
biological indicators									
sex [%]	female	71.4	28.6	19.5	23.0	25.6	32.0	1.9	77.1
	male	71.5	28.5	10.7	18.7	28.5	42.1	1.7	70.3
age [years]	mean	40.1 yrs	54.0 yrs	45.1 yrs	46.4 yrs	44.9 yrs	41.8 yrs		
age [%]	20–29	89.8	10.2	43.8	13.7	17.7	24.8	1.0	79.0
	30–39	84.0	16.0	41.0	14.9	18.4	25.7	1.3	76.3
	40–49	72.3	27.7	32.8	15.4	21.8	30.0	1.8	71.9
	50–59	56.9	43.1	33.4	17.2	24.0	25.4	2.4	70.3
	60–69	49.5	50.5	30.9	16.9	23.8	28.4	2.6	69.8
	70+	40.11	59.9	29.6	15.0	25.8	29.6	2.8	69.1
race/ethnicity [%]	non-Hispanic White	71.5	28.5	9.3	18.5	28.6	43.6	1.8	79.3
	non-Hispanic Black	67.6	32.4	49.4	27.8	15.0	7.8	1.9	48.3
	Mexican-American	72.0	28.0	22.3	29.2	28.3	20.2	1.8	62.8
	other	75.1	24.9	24.9	30.4	25.8	18.9	1.7	61.5
socioeconomic indicators									
education (missing 0.4%) [%]	less than high-school	60.6	39.4	17.2	23.1	27.2	32.6	2.2	71.0
	high-school graduate	69.7	30.3	16.8	20.9	25.4	36.9	1.8	72.9
	college graduate	79.0	21.0	12.8	19.7	28.2	39.3	1.5	76.3
census region [%]	northeast	72.4	27.6	10.5	18.4	27.3	43.8	1.7	79.2
	midwest	71.5	28.5	11.1	17.7	28.1	43.2	1.8	79.0
	south	69.0	31.0	19.7	21.7	25.5	33.1	1.9	70.2
	west	74.4	25.6	17.2	25.9	28.0	28.9	1.7	68.8
urbanisation [%]	urban environment	74.4	25.6	18.6	23.2	26.5	31.7	1.7	69.9
	rural environment	68.6	31.4	12.0	18.8	27.5	41.8	1.9	77.7
marital status (missing 0.1%) [%]	married and living as married	70.6	29.4	12.8	20.5	27.5	39.2	1.8	75.6
	widowed, divorced or separated	61.1	38.9	22.2	23.2	25.5	29.2	2.1	68.2
	never married	86.0	14.0	17.7	20.4	26.3	35.6	1.2	72.9
poverty income ratio (missing 5.8%) [%]	below poverty	65.8	34.2	23.4	22.1	25.4	29.1	2.0	68.0
	at or above poverty	72.7	27.3	14.1	20.6	27.3	38.0	1.7	74.8
lifestyle indicators									
alcohol consumption [%]	non-drinker	63.0	37.0	17.6	23.7	25.5	33.2	2.1	70.6
	moderate-drinker	78.2	21.8	13.3	18.6	28.4	39.7	1.5	76.5
	excessive-drinker	75.6	24.4	15.1	20.9	25.0	39.1	1.6	75.0
smoking status [%]	non-smoker	74.6	25.4	15.8	21.9	27.8	34.5	1.6	72.7
	current-smoker	72.9	27.1	17.1	20.0	25.0	37.8	1.8	74.2
	ex-smoker	64.3	35.7	12.1	20.3	27.7	39.9	2.1	75.7
physical activity (moderate & vigorous) [%]	no activities	63.0	37.0	25.2	25.7	25.4	23.7	2.1	64.0
	1–2 activities per week	69.1	30.9	17.1	23.7	26.9	32.3	1.9	70.8
	3–4 activities per week	74.4	25.6	9.9	20.5	30.9	38.8	1.7	76.0
	5+ activities per week	75.8	24.2	11.1	17.3	26.6	45.0	1.6	79.6
diet (healthy eating index) (missing 2.9%)	mean (1–100)	63.5 pts	64.1 pts	61.4 pts	63.7 pts	64.4 pts	64.0 pts		
diet [%]	healthy eater	69.4	30.6	37.7	12.4	21.9	28.0	1.8	74.5
	unhealthy eater	73.0	27.0	36.7	16.9	20.2	26.2	1.7	73.7
health indicators									
self-reported health status [%]	excellent	84.9	15.1	11.5	18.5	27.7	42.3	1.3	78.9
	very good and good	71.3	28.7	15.1	20.3	27.1	37.5	1.8	74.1
	fair and poor	53.3	46.7	21.0	27.2	25.4	26.4	2.5	65.9
long-term medication [%]	no medication	79.3	20.7	14.3	19.9	27.3	38.5	1.5	75.1
	medication	46.2	53.8	18.2	24.2	26.2	31.5	3.0	69.1

AL, allostatic load; 25(OH)D, 25-hydroxyvitamin D concentration in quartiles.

Vitamin D concentrations were significantly associated with AL (Table 3). For all four AL versions tested and for all adjustments, the odds ratios for high AL were statistically significantly higher for participants in the lowest vitamin D quartile compared to the highest quartile. In the fully adjusted model, the ORs of having high AL for those in the lowest 25(OH)D quartile were 1.45 (for AL1 quartiles: 95% CI: 1.28,1.67) and 1.79 (for AL2 cutpoints: 95% CI: 1.39, 2.32) depending on AL definition.

**Table 3:** Regression results for AL and 25(OH)D concentration.

**Logistic Regression (odds ratio (95% confidence interval))**						
		**Vitamin D in Quartiles**				**Vitamin D Continuous**
**Endpoint**	**Model**	**Q1**	**Q2**	**Q3**	**Q4**	**per 10nmol/L**
AL1 cut points	basic	1.86 (1.67, 2.08)	1.75 (1.59, 1.94)	1.25 (1.14, 1.38)	1.00	0.91 (0.90, 0.93)
	biological	1.62 (1.43, 1.85)	1.45 (1.30, 1.62)	1.08 (0.97, 1.20)	1.00	0.94 (0.93, 0.96)
	socioeconomic	1.78 (1.55, 2.04)	1.52 (1.36, 1.71)	1.15 (1.04, 1.29)	1.00	0.93 (0.92, 0.95)
	lifestyle	1.59 (1.38, 1.83)	1.41 (1.25, 1.59)	1.13 (1.01, 1.26)	1.00	0.95 (0.93, 0.96)
	full	1.53 (1.32, 1.77)	1.38 (1.22, 1.56)	1.15 (1.02, 1.29)	1.00	0.95 (0.94, 0.97)
AL1 quartiles	basic	2.25 (1.92, 2.63)	1.96 (1.70, 2.25)	1.38 (1.21, 1.59)	1.00	0.89 (0.88, 0.91)
	biological	1.86 (1.53, 2.25)	1.48 (1.27, 1.74)	1.12 (0.96, 1.31)	1.00	0.93 (0.91, 0.95)
	socioeconomic	1.88 (1.53, 2.30)	1.49 (1.26, 1.76)	1.16 (0.99, 1.37)	1.00	0.93 (0.91, 0.95)
	lifestyle	1.63 (1.32, 2.03)	1.34 (1.13, 1.60)	1.12 (0.94, 1.32)	1.00	0.95 (0.93, 0.98)
	full	1.59 (1.27, 1.98)	1.33 (1.11, 1.59)	1.14 (0.96, 1.35)	1.00	0.95 (0.93, 0.98)
AL2 cut points	basic	2.81 (2.35, 3.38)	2.67 (2.28, 3.14)	1.65 (1.43, 1.92)	1.00	0.86 (0.84, 0.87)
	biological	2.23 (1.79, 2.78)	1.98 (1.65, 2.37)	1.36 (1.16, 1.60)	1.00	0.89 (0.87, 0.92)
	socioeconomic	2.22 (1.75, 2.83)	2.09 (1.72, 2.53)	1.44 (1.21, 1.71)	1.00	0.89 (0.86, 0.91)
	lifestyle	1.86 (1.45, 2.40)	1.88 (1.54, 2.31)	1.31 (1.09, 1.57)	1.00	0.91 (0.88, 0.93)
	full	1.88 (1.46, 2.43)	1.89 (1.54, 2.31)	1.27 (1.06, 1.53)	1.00	0.91 (0.88, 0.93)
AL2 quartiles	basic	2.71 (2.24, 3.28)	1.98 (1.68, 2.32)	1.53 (1.32, 1.77)	1.00	0.87 (0.85, 0.89)
	biological	2.06 (1.65, 2.60)	1.42 (1.19, 1.70)	1.26 (1.07, 1.48)	1.00	0.91 (0.89, 0.93)
	socioeconomic	1.87 (1.47, 2.40)	1.34 (1.11, 1.63)	1.32 (1.11, 1.58)	1.00	0.92 (0.89, 0.94)
	lifestyle	1.59 (1.23, 2.05)	1.22 (1.00, 1.49)	1.23 (1.03, 1.48)	1.00	0.93 (0.91, 0.96)
	full	1.61 (1.24, 2.08)	1.22 (0.99, 1.49)	1.20 (1.00, 1.45)	1.00	0.93 (0.91, 0.96)
**Poisson Regression (rate ratio (95% confidence interval))**						
		**Vitamin D in Quartiles**				**Vitamin D Continuous**
**Endpoint**	**Model**	**Q1**	**Q2**	**Q3**	**Q4**	**per 10nmol/L**
AL1 cut points	basic	1.29 (1.24, 1.34)	1.26 (1.21, 1.3)	1.12 (1.08, 1.16)	1.00	0.96 (0.96, 0.97)
	biological	1.19 (1.15, 1.24)	1.14 (1.10, 1.18)	1.05 (1.02, 1.08)	1.00	0.98 (0.97, 0.98)
	socioeconomic	1.21 (1.16, 1.26)	1.15 (1.11, 1.19)	1.06 (1.03, 1.10)	1.00	0.97 (0.97, 0.98)
	lifestyle	1.16 (1.11, 1.21)	1.12 (1.08, 1.16)	1.05 (1.02, 1.09)	1.00	0.98 (0.97, 0.98)
	full	1.15 (1.10, 1.20)	1.11 (1.07, 1.15)	1.05 (1.02, 1.09)	1.00	0.98 (0.98, 0.99)
AL1 quartiles	basic	1.41 (1.33, 1.50)	1.35 (1.28, 1.42)	1.15 (1.09, 1.21)	1.00	0.95 (0.94, 0.96)
	biological	1.24 (1.17, 1.32)	1.17 (1.11, 1.23)	1.04 (0.99, 1.10)	1.00	0.97 (0.96, 0.98)
	socioeconomic	1.24 (1.17, 1.32)	1.16 (1.10, 1.23)	1.05 (1.00, 1.11)	1.00	0.97 (0.97, 0.98)
	lifestyle	1.17 (1.10, 1.25)	1.12 (1.06, 1.18)	1.04 (0.98, 1.09)	1.00	0.98 (0.97, 0.99)
	full	1.15 (1.08, 1.23)	1.11 (1.06, 1.18)	1.04 (0.99, 1.09)	1.00	0.98 (0.97, 0.99)
AL2 cut points	basic	1.45 (1.37, 1.54)	1.41 (1.34, 1.48)	1.19 (1.13, 1.25)	1.00	0.94 (0.94, 0.95)
	biological	1.30 (1.22, 1.38)	1.25 (1.19, 1.31)	1.10 (1.05, 1.16)	1.00	0.96 (0.95, 0.97)
	socioeconomic	1.30 (1.22, 1.38)	1.25 (1.18, 1.31)	1.12 (1.07, 1.18)	1.00	0.96 (0.95, 0.97)
	lifestyle	1.24 (1.16, 1.33)	1.21 (1.15, 1.28)	1.09 (1.04, 1.15)	1.00	0.96 (0.96, 0.97)
	full	1.23 (1.15, 1.31)	1.20 (1.14, 1.27)	1.09 (1.03, 1.14)	1.00	0.97 (0.96, 0.97)
AL2 quartiles	basic	1.44 (1.36, 1.53)	1.37 (1.30, 1.45)	1.18 (1.12, 1.24)	1.00	0.95 (0.94, 0.95)
	biological	1.28 (1.21, 1.36)	1.22 (1.16, 1.28)	1.09 (1.04, 1.14)	1.00	0.96 (0.95, 0.97)
	socioeconomic	1.27 (1.19, 1.35)	1.21 (1.15, 1.27)	1.11 (1.05, 1.16)	1.00	0.96 (0.96, 0.97)
	lifestyle	1.21 (1.14, 1.29)	1.17 (1.11, 1.23)	1.08 (1.02, 1.13)	1.00	0.97 (0.96, 0.98)
	full	1.20 (1.13, 1.28)	1.16 (1.10, 1.22)	1.07 (1.02, 1.12)	1.00	0.97 (0.96, 0.98)

For logistic regression AL, allostatic load, was included as a binary variable (high or low). For Poisson regression AL, allostatic load, was included as count of the numbers of biomarkers at risk; 25(OH)D, serum 25-hydroxyvitamin D concentration. AL was the only variable considered for the basic, unadjusted model. As „biological”factors age, sex and race/ethnicity were included, “socioeconomic” variables comprised education, census region, urbanization, marital status, poverty-income ratio, “lifestyle” factors alcohol consumption, smoking status, physical activity, diet. Additionally, self-reported general health was added to the full model.

Considering vitamin D concentration as a continuous variable showed similar results. In all cases, increasing vitamin D by 10 nmol/L was slightly but statistically significantly associated with a decrease in allostatic load (Table 3), with ORs ranging from 0.91 (for AL2 cutpoint: 95% CI: 0.88, 0.93) to 0.95 (for AL1 cutpoint: 95% CI: 0.94, 0.97; for AL1 quartile, 95% CI: 0.93, 0.98).

Examining allostatic load as the count of the corresponding biomarkers, subjects with the lowest quartile vitamin D had up to 1.23 times the AL score as those in the highest quartile (rate ratio for AL2 cutpoint, 95% CI: 1.15–1.31) (Table 3). Further considering vitamin D as a continuous covariate, vitamin D continues to show a slight but statistically significant negative association with allostatic load, with a rate ratio for AL1 cutpoint of 0.98 (95% CI: 0.98–0.99). Results from the linear regression analysis of allostatic load are found in the supplementary material (S1 Table).

**Fig 2:**
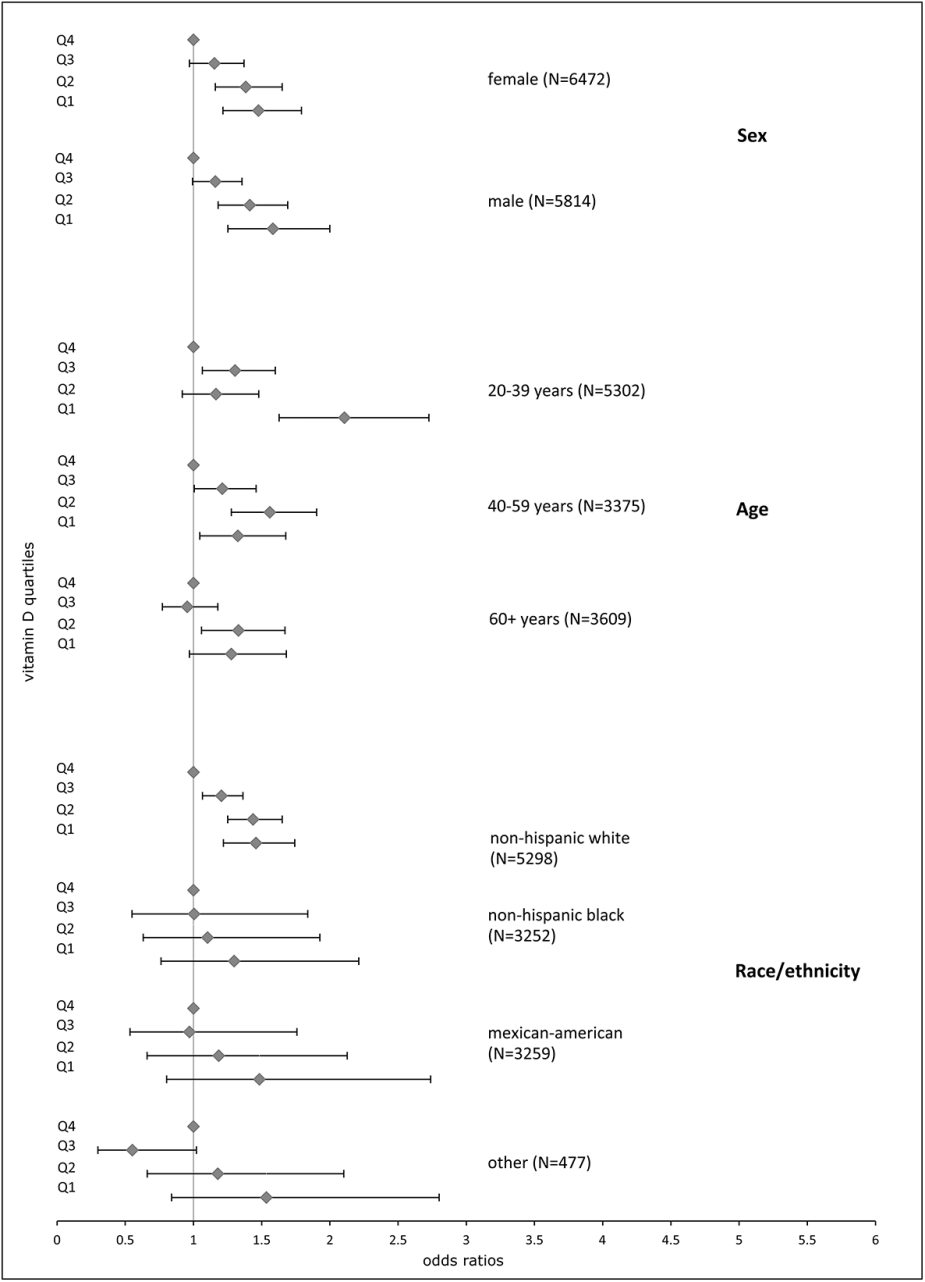
Logistic regression analysis of the allostatic load index and 25(OH)D concentrations stratified by biological covariates. Results are stratified by sex, age, and ethnic background and are presented as ORs for high AL with 95% confidence intervals. Q, 25(OH)D concentrations in quartiles with Q1 lowest and Q4 highest quartile (reference).

Stratifyingthe analysis by sex showed no differences between male and female participants (Fig 2). No cut-off age that altered the relationship of vitamin D with AL was observed when logistic regression was performed by age categories, although sensitivity to low 25(OH)D concentrations was highest among the youngest group (20–39 years) with an OR of 2.11 (95% CI: 1.63–2.73) for the lowest vitamin D quartile Q1. Higher serum 25(OH)D was associated with lower AL among all ethnicities, but only non-Hispanic Whites showed significant ORs for all vitamin D quartiles compared to the highest quartile, with an OR of 1.46 for Q1 (95% CI: 1.22–1.74). In all other ethnic groups, the comparison between top versus bottom quartile was statistically insignificant.

Among participants describing their general health as “excellent”, we observed no statistically significant association between low 25(OH)D concentration and low AL, with an OR of 0.66 (95% CI: 0.42; 1.04) for Q1. However, participants considering their general health as “very good and good” or “fair and poor”showed significant associations between low 25(OH)D concentrations and high AL.

**Fig 3:**
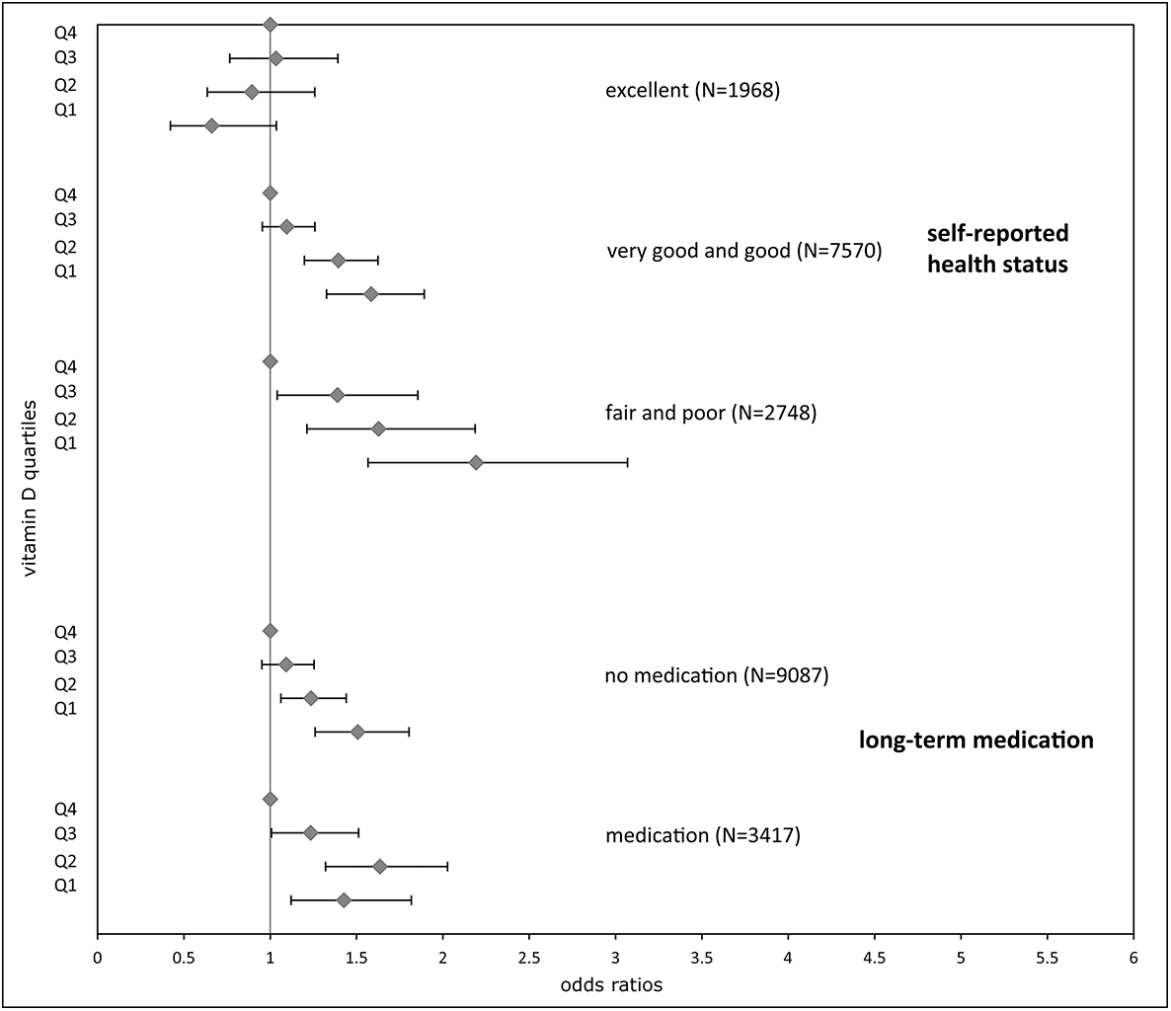
Logistic regression analysis of the allostatic load index and 25(OH)D concentrations stratified by health covariates. Results are stratified by self-reported health status, and long-term medication and are presented as ORs for high AL with 95% confidence intervals. Q, 25(OH)D concentrations in quartiles with Q1 lowest and Q4 highest quartile (reference).

When logistic regression was performed with long-term medication, this phenomenon did not recur as participants with no medication had an OR of 1.51 (95%CI: 1.26; 1.80) for Q1 (Fig 3). Adding single medication groups (such as antihypertensives, antihyperlipidemics, blood glucose regulators, mental health medications, and antineoplastics) instead of the combination of all of them did not change the results significantly. Also, adding vitamin D supplementation to the fully adjusted logistic regression model did not change ORs significantly (results not shown).

## Discussion

In this cross-sectional study, we observed a statistically significant and consistent inverse relationship between serum 25(OH)D concentration and AL as a measure of cumulative biological risk. Various ways of assessing AL provided consistent results. Those results remained similar when the analyses were adjusted for biological, socioeconomic status, lifestyle and general health status. This is the first report examining the association of serum vitamin D concentrations and AL.

The observed relationship could be explained by the various functions that vitamin D has on immune homeostasis, and there is increasing evidence that higher serum 25(OH)D concentrations can alleviate inflammatory diseases. Our findings are consistent with the results of other studies showing an inverse association of serum 25(OH)D concentrations with health events or biomarkers we had included in our AL index, including obesity [39], hypertension [40, 41], insulin resistance [42–44], CRP concentrations [45], and dyslipidemia [46]. Weyland et al. reviewed the evidence to support a causal association between vitamin D status and cardiovascular disease risk according to Hill’s criteria and found that all relevant criteria for causal association were fulfilled [47]. However, an umbrella review of hundreds of studies showed that results differ between observational and intervention studies such, that the causal effect of vitamin D on mortality remains unclear [22]. The same conclusion needs to be drawn for e.g. cardiovascular diseases, cancer, or diabetes mellitus.

The association of vitamin D and AL remained consistent after stratification by several biological and health status variables. That females had a significantly lower mean 25(OH)D serum concentration than males did not affect the association between vitamin D and AL.

Vulnerability to high AL has been linked to minority ethnic status by many authors [16, 48], which we reproduced in our study (Table 2). We observed significantly higher 25(OH)D concentrations in non-Hispanic Whites (79.1 nmol/L) compared to non-Hispanic Blacks (48.2 nmol/L) and Mexican-Americans (62.6 nmol/L). This is mostly due to different effectiveness of vitamin D synthesis depending on skin pigmentation, but other factors, such as distinct outdoor activities or dietary patterns may contribute to these differences as well. However, we only observed statistically significant associations for non-Hispanic Whites (Fig 2).

Within our sample, the prevalence of a high AL was 13.2% for participants aged 20–39 years, 33.8% for 40–59 year aged and as high as 54.9% for participants older than 60 years. It is well known that AL increases (non-linearly) with age, which is plausible because AL measures the cumulative biological risk that increases with age [49]. Sensitivity to low 25(OH)D concentrations was highest among the youngest group (20–39 years) with an OR of 2.11 (95% CI: 1.63; 2.73) for the lowest vitamin D quartile Q1, but higher 25(OH)D concentrations were associated with lower OR for high AL within all age categories. By including all adult age groups in the analysis we were able to identify an otherwise neglected at-risk group, those aged 20–39 years. Further research is needed to understand the relevance of this finding for this age group.

Self-reported general health has been shown to be a reliable predictor for mortality, even when controlling for other health-related variables [50, 51] and a recent study showed an association with AL [52]. Among the different health dimensions, vitality, in particular,was important for individuals to assess their self-reported general health [53]. Interestingly, for participants who considered their health status as “excellent,” no statistically significant association between higher 25(OH)D concentrations and AL was observed (Fig 3).

When long-term medication was used as an indicator of health (instead of self-reported general health), this effect did not appear for participants that took no medication; instead we observed a significant relationship between high vitamin D concentrations and AL.

We tested four different AL versions because there are no accepted or validated standards for this practice (number and type of biomarkers to be included, method of dichotomization). The AL1 cut point is equivalent to the method used in several current studies [8, 14, 17, 21]. Quartiles instead of cut points were used for the dichotomization of the biomarkers [48], to account for an internal reference instead of external ones. To investigate the impact of additional biomarkers, we added BMI, TG, H1A, H2A and serum creatinine to both models. Thereby, we were consistent with other studies and were able to: allow for comparisons (BMI, TG), target supplemental markers of the immune system (H1A, H2A) as well as the cardio-renal system and control for a potential relationship between renal function and serum 25(OH)D concentrations [54]. We decided not to include weights for each factor as there are no standards for that.

ORs for high AL between the four tested versions were rather similar (Table 3). However, the AL2 cut point differed in terms of gradual reduction of OR from Q4 to Q1, as it showed very similar values for Q1 and Q2 and a drop-off to Q3, which were explained by TG and creatinine. Despite this, a significant difference between the cut point and the quartile risk categorization was observed when looking at individual biomarkers; in the cut point model, a higher proportion of about 25% of the participants was allocated to the high-risk category. For the specific selection of biomarkers studied here, these differences cancelled each other out when summed to the AL index. However, for another selection of biomarkers this may not necessarily be the case, and other methods of risk classification could lead to different outcomes.

In the current study neither the number of biomarkers nor the method of dichotomization changed the results in a significant way. Due to the exclusion of participants with missing values for one of the biomarkers, the sample size was reduced from 14,213 participants for AL1 to 4,620 for AL2, which made AL2 the weaker tool for the statistical analyses of subgroups. Additional modeling as a further validity measure, such as examining the vitamin D concentration as a continuous variable and analyzing the allostatic load as a count of the corresponding biomarkers, provided the same statistically significant inverse relationship between vitamin D and AL.

The lack of agreement among prior studies on the inclusion of different biomarkers makes it difficult to compare results of different studies. Two recent reviews on measuring AL identified a total of 39 and 51different biomarkers, respectively, that were used to calculate the AL index [12, 13]. Further studies are needed to examine which combination of biomarkers represents the most valid and robust aggregate predictor of adverse health outcomes. We provide some evidence to include 25(OH)D concentrations in AL scores, but further assessments are needed.

Furthermore, AL, self-reported general health and vitamin D concentrations represent relevant markers for a health assessment as they are all associated with all-cause mortality. Within this study, their relationships were shown to be statistically significant. It remains to be seen whether preventive measures to influence these parameters might have an effect on one another as well as on health events.

Up to now, evidence that vitamin D supplementation is indicated to treat or prevent several chronic diseases is inconsistent and potential mechanisms are only partially elucidated. In spite of the widespread fortification of various foods with vitamin D, supplementation and supply from sunlight exposure, the prevalence of deficiency increased between 1988–1994 and 2001–2002 and remained on the same concentration until 2005–2006 in US adults [55]. The optimal serum concentration of 25(OH)D is still under debate and no general definitions for deficiencies are available. It might be that for prevention of immune-related diseases, higher vitamin D concentrations are needed than for bone and mineral homeostasis [47, 56]. Furthermore, it is still an open question whether a low 25(OH)D serum concentration is the cause or effect of disease. In the near future, results of four new long-term and large-scale supplementation studies are expected on the effect of vitamin D on cancer, cardiovascular diseases and diabetes, which will hopefully fill some of the current knowledge gaps [57].

This study provides no knowledge about the causal relationship of serum 25(OH)D concentrations and AL due to its cross-sectional design. 25(OH)D is considered indicative of an individual’s vitamin D status, whereas 1,25(OH)_2_D, the active metabolite, is homeostatically controlled in the blood [58]. Thus, only 25(OH)D, but not 1,25(OH)2D concentrations, have been taken into account and the latter could have a cross-regulating effect [30].

Our study has several strengths. The study population is nationally representative for non-institutionalized US adults, which, therefore, enhances the generalizability of the results. The inclusion of a wide variety of biological, demographic, socioeconomic status, lifestyle, and health status-associated covariates reduces the risk of confounding. To our knowledge, AL has not previously been assessed with such a broad spectrum of covariates. The study contributes to a limited body of research on the relationship of cumulative stress exposure, AL as a health risk indicator, and vitamin D status.

## Conclusions

Allostatic load is a powerful tool to assess the harmful effects of chronic stress to the human body on a multisystem level. There is an urgent need of agreement on the method and the biomarkers to calculate the AL index. Vitamin D has a statistically significant inverse association with all tested models of high AL, which was persistent after adjusting for biological, socioeconomic, lifestyle and health variables. Our study adds to the increasing evidence linking low vitamin D serum concentrations to adverse health outcomes, beyond its role in bone health. Prospective studies need to address the question whether low 25(OH)D concentrations are the cause or the outcome of high AL. If vitamin D supplementation can be shown to lower AL, vitamin D could become a relevant preventive measure to reduce the harmful effects of chronic stress in humans.

## Supporting Information

S1 Table Linear regression results for allostatic load (AL) and 25-hydroxy-vitamin D (vitamin D). (DOCX)
