# Supplementary material for: Relationship of Serum Vitamin D Concentrations and Allostatic Load as a Measure of Cumulative Biological Risk among the US Population: A Cross-Sectional Study
Source: PLoS One. 2015 Oct 9;10(10):e0139217. doi: 10.1371/journal.pone.0139217 (PMC4599851; doi:10.1371/journal.pone.0139217)
Supplement: S1 Table — (DOCX) [file pone.0139217.s001.docx]

Relationship of Serum Vitamin D Concentrations and Allostatic Load as a Measure of Cumulative Biological Risk among the US Population: a Cross-Sectional Study

Supplementary information

**S1 Table. Linear regression results for allostatic load (AL) and 25-hydroxy-vitamin D (vitamin D).**

| **Linear Regression (mean difference (95% confidence interval))** | | | | | | |
| --- | --- | --- | --- | --- | --- | --- |
|  |  | **Vitamin D in Quartiles** | | | | **Vitamin D Continuous** |
| **Endpoint** | **Model** | **Q1** | **Q2** | **Q3** | **Q4** | **per 10nmol/L** |
| AL1 cut points | basic | 0.45 (0.38, 0.53) | 0.41 (0.34, 0.47) | 0.19 (0.13, 0.25) | 0.00 | -0.07 (-0.07, -0.06) |
|  | biological | 0.30 (0.23, 0.37) | 0.22 (0.16, 0.28) | 0.06 (0.01, 0.12) | 0.00 | -0.04 (-0.04, -0.03) |
|  | socioeconomic | 0.31 (0.24, 0.39) | 0.23 (0.16, 0.29) | 0.08 (0.02, 0.14) | 0.00 | -0.04 (-0.05, -0.03) |
|  | lifestyle | 0.23 (0.15, 0.30) | 0.17 (0.11, 0.23) | 0.06 (0.00, 0.11) | 0.00 | -0.03 (-0.04, -0.02) |
|  | full | 0.20 (0.12, 0.27) | 0.15 (0.09, 0.21) | 0.06 (0.00, 0.11) | 0.00 | -0.02 (-0.03, -0.02) |
| AL1 quartiles | basic | 0.68 (0.56, 0.80) | 0.58 (0.47, 0.69) | 0.25 (0.15, 0.35) | 0.00 | -0.09 (-0.11, -0.08) |
|  | biological | 0.41 (0.29, 0.53) | 0.26 (0.17, 0.36) | 0.04 (-0.05, 0.13) | 0.00 | -0.04 (-0.06, -0.03) |
|  | socioeconomic | 0.39 (0.26, 0.52) | 0.24 (0.14, 0.34) | 0.06 (-0.04, 0.15) | 0.00 | -0.04 (-0.05, -0.03) |
|  | lifestyle | 0.28 (0.15, 0.40) | 0.16 (0.05, 0.26) | 0.02 (-0.07, 0.12) | 0.00 | -0.02 (-0.04, -0.01) |
|  | full | 0.24 (0.11, 0.37) | 0.14 (0.04, 0.25) | 0.03 (-0.06, 0.12) | 0.00 | -0.02 (-0.04, -0.01) |
| AL2 cut points | basic | 1.14 (0.96, 1.31) | 1.02 (0.86, 1.18) | 0.48 (0.33, 0.62) | 0.00 | -0.16 (-0.18, -0.14) |
|  | biological | 0.77 (0.59, 0.95) | 0.62 (0.47, 0.77) | 0.23 (0.09, 0.37) | 0.00 | -0.11 (-0.12, -0.09) |
|  | socioeconomic | 0.74 (0.55, 0.93) | 0.60 (0.44, 0.75) | 0.27 (0.13, 0.41) | 0.00 | -0.11 (-0.13, -0.09) |
|  | lifestyle | 0.60 (0.40, 0.79) | 0.50 (0.34, 0.66) | 0.20 (0.05, 0.34) | 0.00 | -0.09 (-0.11, -0.07) |
|  | full | 0.57 (0.37, 0.76) | 0.48 (0.32, 0.64) | 0.18 (0.04, 0.32) | 0.00 | -0.08 (-0.11, -0.06) |
| AL2 quartiles | basic | 1.23 (1.04, 1.43) | 1.04 (0.87, 1.22) | 0.49 (0.33, 0.66) | 0.00 | -0.18 (-0.20, -0.15) |
|  | biological | 0.79 (0.58, 0.99) | 0.60 (0.43, 0.76) | 0.22 (0.07, 0.37) | 0.00 | -0.11 (-0.13, -0.09) |
|  | socioeconomic | 0.73 (0.52, 0.94) | 0.54 (0.37, 0.72) | 0.25 (0.09, 0.41) | 0.00 | -0.10 (-0.12, -0.08) |
|  | lifestyle | 0.57 (0.35, 0.79) | 0.42 (0.25, 0.60) | 0.17 (0.01, 0.33) | 0.00 | -0.08 (-0.10, -0.06) |
|  | full | 0.54 (0.32, 0.75) | 0.40 (0.23, 0.57) | 0.15 (-0.01, 0.30) | 0.00 | -0.08 (-0.10, -0.05) |

AL, allostatic load as continuous variable; 25(OH)D, serum 25-hydroxyvitamin D concentration.

AL was the only variable considered for the basic, unadjusted model. As „biological“ factors age, sex and race/ethnicity were included, “socioeconomic” variables comprised education, census region, urbanization, marital status, poverty-income ratio, “lifestyle” factors alcohol consumption, smoking status, physical activity, diet. Additionally, self-reported general health was added to the full model.
